# Adverse effects of empathy and cognitive inflexibility on social trauma

**DOI:** 10.3389/fpsyg.2023.1090297

**Published:** 2023-05-26

**Authors:** Shisei Tei, Junya Fujino

**Affiliations:** ^1^Department of Psychiatry, Kyoto University, Kyoto, Japan; ^2^Institute of Applied Brain Sciences, Waseda University, Saitama, Japan; ^3^School of Human and Social Sciences, Tokyo International University, Saitama, Japan; ^4^Medical Institute of Developmental Disabilities Research, Showa University, Tokyo, Japan; ^5^Department of Psychiatry and Behavioral Sciences, Tokyo Medical and Dental University, Tokyo, Japan

**Keywords:** trauma, empathy, flexibility, stigma, distress

## Introduction

The key feature of traumatic events defined in the Diagnostic and Mental Manual of Mental Disorders (DSM) can be threats to life (e.g., exposure to death, serious injury, or violence; American Psychiatric Association, 2013). Researchers argue that additional approaches should be utilized to further evaluate what makes an experience traumatic (Bjornsson et al., 2020; Neuner, 2022). In the case of post-traumatic stress disorder (PTSD) and other disorders such as social anxiety disorder (SAD), different types of threats can play crucial roles in their development (Erwin et al., 2006; Carleton et al., 2011). One such threat is social trauma (Hamburger, 2021).

Social trauma is defined as an individual's experience of being socially humiliated or rejected in interpersonal situations, which can severely endanger one's social integrity (Neuner, 2022). It is also characterized as a threat to societal and cultural groups to explain group-specific fears, which are frequently associated with emotional abuse, bullying, and persecution that can develop into guilt, self-blame, or anger (Hamburger, 2021). People who are exposed to negative social events sometimes perceive, experience, or appraise these events as threats, whereby core social motives can be violated (e.g., the need for social status and belonging), and they are often associated with intense affective reactions, including dread, despair, and defeat. Indeed, a group of individuals may react to a social threat such that they live life as if under a social fear, with accompanying PTSD and SAD symptoms as one integrated condition (Bjornsson et al., 2020). These symptoms include intrusive memories, vigilance, and avoidance of social situations (Neuner, 2022). However, how people perceive societal experiences or life events as social trauma and how they can be mitigated remain insufficiently examined. Thus, advancing our understanding of the psychological mechanisms associated with social trauma is crucial.

**Figure 1:**
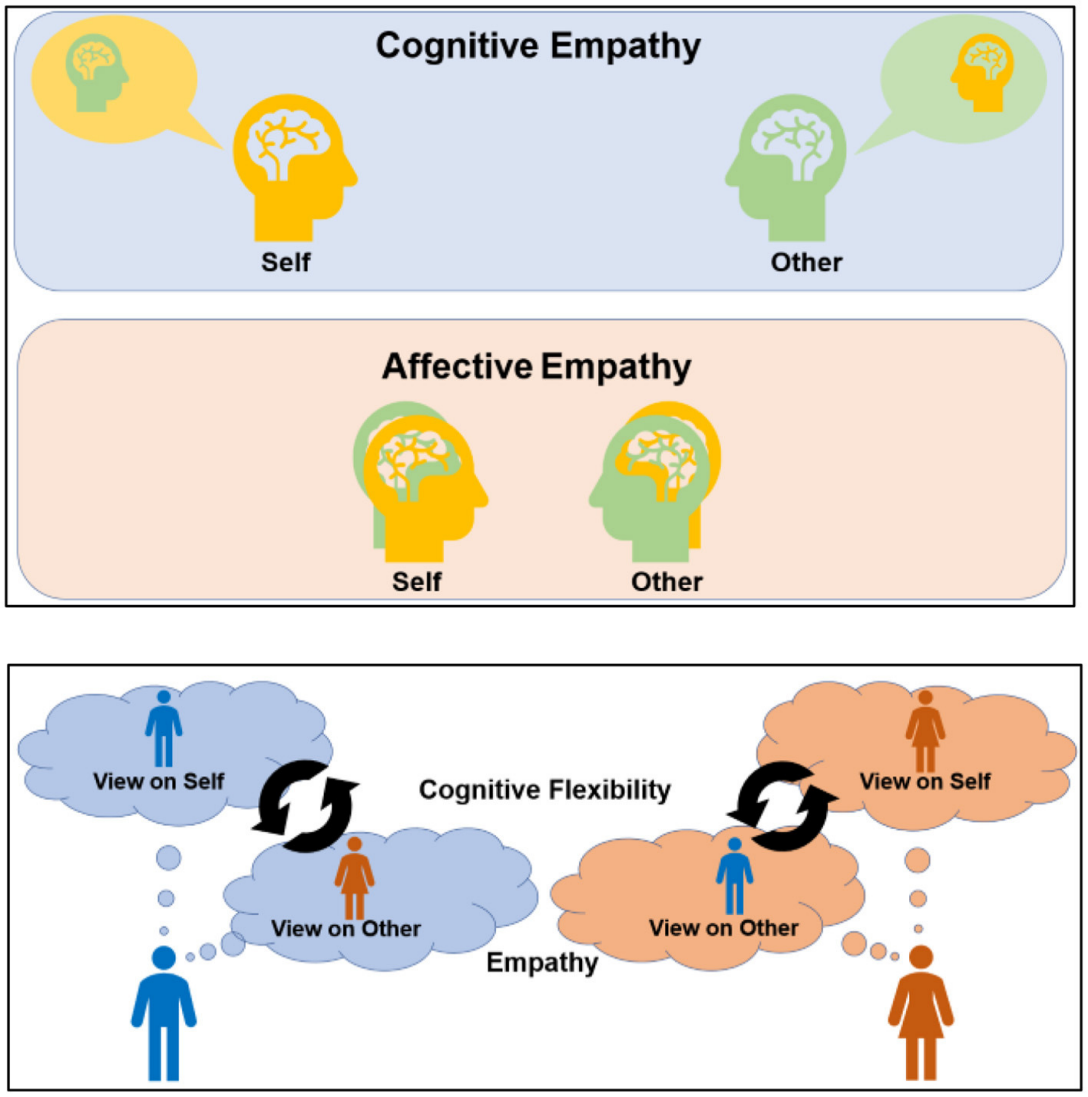
Illustration of empathy and cognitive flexibility. CogEMP allows individuals to obtain accurate knowledge regarding the content of another person's mind and often requires a self–other distinction to identify others' minds more clearly. Meanwhile, AffEMP promotes the sharing of others' feelings, such as joy and distress and involves the self–other overlap of emotional experiences **(upper panels)**. Cognitive flexibility requires self-control to adapt to changing environments via shifts in decision rules and perspectives. It also supports the switching and maintenance of perspectives between the self and others to facilitate empathy, which prompts social communication that is adaptable and context-adjusted **(lower panel)**. Meanwhile, altered cognitive flexibility and empathy may hinder flexible emotion regulation and shifting attention and perspectives. They can maladaptively amplify shared distress and perspective bias (empathic inaccuracy), which can prompt people to acknowledge interpersonal communication or experiences as socially traumatic.

The experience of trauma may be aggravated when empathic responses are not appropriately moderated by the social context (Levy et al., 2019; Couette et al., 2020). Broadly, empathy comprises emotion sharing and mentalizing (Singer and Lamm, 2009; Yu and Chou, 2018) and is essential for social living; however, it may exacerbate certain traumatic experiences (Klimecki and Singer, 2012; Branson, 2019). Maladaptive affective empathy (AffEMP; Figure 1) can prompt a disproportionate affective intrusion of others' distress, which can trigger traumatic experiences, whereas maladaptive cognitive empathy (CogEMP) can augment cognitive bias or incorrect knowledge of the contents of another person's mind (Naor et al., 2020; Hinnekens et al., 2021) and amplify interpersonal fear.

Survivors with PTSD have also shown greater difficulty in inferring others' emotions from hypothetical vignettes and social targets (Nietlisbach et al., 2010). Furthermore, lower CogEMP levels are associated with social trauma (Williford et al., 2016). That is, while CogEMP can help individuals understand others' feelings and thoughts, inaccurate CogEMP can maladaptively augment observers' distress and induce traumatic experiences. It is possible that maladaptive CogEMP can hinder flexible emotional distancing or the management of traumatic events on a cognitive level (Regehr et al., 2002; Chiu et al., 2016; Greenberg et al., 2018).

According to a systematic review (Couette et al., 2020), both affective and cognitive aspects of empathy might be disturbed in individuals with PTSD, which reportedly impairs their ability to predict what others feel, think, or believe (Mazza et al., 2012; Parlar et al., 2014; Levy et al., 2019). Furthermore, different effects of socio-cognitive and socio-affective mechanisms can be associated with traumatic experiences (Trautmann et al., 2022). In this regard, neuroimaging studies have reported a possible dissociation between AffEMPs and CogEMPs among patients with PTSD (e.g., Mazza et al., 2015). Given that the experience of social trauma can also involve excessive sharing and inaccurate recognition of others' distress (Couette et al., 2020; Neuner, 2022), social emotion and cognition can be substantially affected by both AffEMP and CogEMP. They can maladaptively amplify shared distress and perspective bias (empathic inaccuracy) to develop social trauma.

Furthermore, these adverse effects of empathy might be counteracted by cognitive flexibility, which facilitates a shift in perspective between the self and others (Eslinger, 1998; Supplementary Table S1). More specifically, the process of empathy requires cognitive flexibility, which prompts switching and/or maintenance between perspectives of the self and others in a socially adjusted manner. This process may allow for a flexible analysis of others' viewpoints (as well as that of oneself), and wholesome empathy and flexibility may subserve interpersonal understanding and social functioning (Decety, 2011). In other words, wholesome empathy and cognitive flexibility might play crucial roles in hindering social trauma by suppressing maladaptive negative thoughts about oneself and others (Bjornsson et al., 2020; Hamburger, 2021; Neuner, 2022). Indeed, reduced flexible emotion regulations, difficulty in shifting attention/perspectives, and concomitant rumination are reported among individuals with PTSD and SAD (Haruvi-Lamdan et al., 2018; Goodman et al., 2021), and such overlapping mechanisms might partly account for their shared vulnerability in social trauma.

This commentary draws on existing theoretical models and relevant cognitive studies on social trauma to outline possible disturbances in AffEMP, CogEMP, and cognitive inflexibility as potential risk factors. Additionally, it explores these adverse effects in the context of recent disasters, which can facilitate our understanding of social trauma. Consequently, we posit that inappropriate empathy and inflexibility might play a crucial role in the development of interpersonal distress and fear via maladaptive shifting between self–other viewpoints.

## Traumatic stress, empathy, and flexibility

### Alterity of empathy

Recently, people have expressed feelings of fear, anxiety, and rejection when confronted with and witnessing the war (Avramchuk et al., 2022; Jawaid et al., 2022; Spiegel, 2022), frequently by sharing others' distress via AffEMP. Prolonged or repeated exposure to others' distress, helplessness, and humiliation in societal and cultural contexts can develop into social trauma (Hamburger, 2021; Neuner, 2022). CogEMP can further aggravate social trauma when faced with significant stigma, wherein perspective-taking becomes imprecise (i.e., empathic inaccuracy; Zaki et al., 2009; Ickes and Hodges, 2013). For instance, observing and inferring others' hatred and distress toward one's ethnicity or identity can amplify fear and anxiety, leading to social trauma (Bjornsson et al., 2020).

These negative impacts of CogEMP, whether excessive or insufficient, have also been reported concerning COVID-19. In addition to empathic or shared distress, as stated above, some people manifest interpersonal fear; they express an exaggerated sense of guilt or self-blame in others' eyes. In the earlier phases of the COVID-19 pandemic, observation and attention toward others may have increased for many individuals, particularly in some East Asian cultures, where interpersonal relationships are strongly valued and public errors and disturbances are avoided. Although the development of such other-oriented empathic behaviors may have helped alleviate the pandemic (e.g., wearing masks), some individuals disproportionately feared infecting other people (besides fearing infection; Griffiths and Mamun, 2020). In these experiences, inferring others' thoughts or feelings about oneself may have been inaccurate (Montemurro, 2020; Tei and Wu, 2021).

As such, some people became preoccupied with how others evaluated them, further enhancing negative or biased views of themselves. During the initial phase of COVID-19, especially among people who were infected or quarantined, such experiences were accompanied by guilt and imaginary shame, wherein peer pressure appeared to be augmented in the form of fear. Several individuals amplified traumatic fear via perceived and imaginary stigmas (Sahoo et al., 2020). Some COVID-19-related suicides reported globally, regardless of ethnicity, were associated with the fear of infecting others or others' criticism (Griffiths and Mamun, 2020; Tei and Fujino, 2022a). In this regard, COVID-19-related fear was also linked to beliefs about responsibility and/or trends in obsessive-compulsive symptoms among the general population (Mesterelu et al., 2021).

Incidentally, while empathy-oriented social fear and related traumatic experiences are generally more common in collectivistic cultures and adolescents (Ellis et al., 2020; Magson et al., 2021), recent crises have indicated that they can arise in a wide range of people, regardless of their cultural background. In this regard, the fear of offending others has been specified as one of the primary features of SAD in the DSM-5 (Furukawa, 2014). Relatedly, many refugees have faced the risk of developing social anxiety and concomitant mental problems because of exposure to stigma-related incidents (e.g., hate speech; Bajaj and Stanford, 2022; Knights et al., 2022; Wypych and Bilewicz, 2022). Furthermore, some people have experienced extreme reactions following exposure to social media (e.g., anger and disgust; Cricenti et al., 2022). An increase in this type of empathy-related distress is associated with experiential avoidance and reduced flexible social cognition, which also entails social trauma (Hamburger, 2021; Neuner, 2022).

### Cognitive flexibility

Cognitive flexibility can reduce social trauma, empathy-related shared distress, and concomitant negative experiences (Fu and Chow, 2017; Garner and Golijani-Moghaddam, 2021; Tei and Fujino, 2022a,b). Flexibility prompts a switching of cognitive sets to adapt to a changing social environment and helps refine social cognition by shifting and balancing perspectives about the self and others (Eslinger, 1998; Figure 1). This involves understanding and inferring one's own and others' mental states (including the evaluation or impression of oneself) and becoming aware that they may be different (Decety, 2011; Tei et al., 2020). Specifically, flexibility supports shifting attention between different conflicting perspectives or decision rules, thinking about these perspectives simultaneously, and illuminating alternative viewpoints. Such situation-adjusted responses can moderate traumatic experiences by diverting individuals from a particular perspective, related distress, and self-blame (Zimmer-Gembeck, 2021; Wojcik et al., 2022).

As such, cognitive flexibility can help individuals acknowledge themselves as contextual, relational, and transient (Supplementary Table S1). Indeed, those with greater flexibility are likely to view stressful situations more objectively, feel less attached to a particular belief, and reduce their persistent focus on others' evaluations (Burton et al., 2012). A more flexible or reasonable recognition of the self in relation to others may reduce misunderstandings, fear-triggered bias, and maladaptive distress appraisals. Consequently, cognitive flexibility may alleviate experience of social trauma by modulating the negative effects of empathy (i.e., empathic distress and empathic inaccuracy). However, the validity of this model requires further investigation and refinement. For example, empathy-oriented fear might emerge differently in people with divergent socioeconomic statuses (e.g., Shah, 2007).

## Discussion

This article highlighted the potential adverse effects of empathy and cognitive inflexibility on social trauma. Traumatic experiences emerging from excessive sharing or dealing with others' distress may have recently become more prevalent. In addition to other risk factors (e.g., Lynn et al., 2022), it is possible that both affective and cognitive accounts of empathy may be associated with the experience of social trauma by amplifying observers' distress (Couette et al., 2020; Neuner, 2022).

Such adverse empathy effects can be mitigated by cognitive flexibility, which facilitates recontextualizing the self in relation to others and the social environment. This may develop individuals' awareness of their interpersonal situations more precisely and objectively. Contrarily, those with reduced cognitive flexibility may be at greater risk of developing related psychopathological symptoms. That is, reduced flexibility and inappropriate empathic sensitivity can distort or impact the sharing and recognition of others' viewpoints. This may be associated with the irrational belief that one is responsible for others' distress and stigma, which can augment interpersonal fear or social trauma (Bjornsson et al., 2020; Neuner, 2022). Accordingly, investigating whether social trauma involves altered stress signaling pathways in those who exaggerate others' distress or maladaptively deal with stigma seems worthwhile.

Further studies on social trauma and its underlying cognitive mechanisms can deepen our understanding of trauma by extending the current psychological models of PTSD (e.g., Ehlers and Clark, 2000). Although the empathy–flexibility model remains provisional, and evidence supporting its mental processes and cognitive mechanisms beyond existing hypotheses is limited, we believe that the proposed trajectory will provide useful targets for clinical interventions and future empirical studies. For example, investigating the possible interactive and reciprocal nature of flexibility, AffEMP, and CogEMP as well as how they affect the concomitant traumatic experience are worthwhile. Confirming their unique neurocognitive mechanisms and identifying other associated social and psychological factors may improve the prediction of unfavorable reactions to social trauma. Empathy is a multidimensional process (Coll et al., 2017) in which various facets can distort social cognition. With increasing humanitarian and natural disasters, monitoring and investigating variations in fear-related experiences and exploring the societal and individual determinants of stigmatization are crucial (e.g., susceptibility to shame, proneness to empathy, and reduced cognitive flexibility). In terms of social trauma, whether repeated exposure to stigma (perceived, imagined, or anticipated) and exaggerated empathic distress could develop into stress injuries as another type of mass trauma remains a major concern. We hope that this commentary will stimulate discussion and motivate more comprehensive empirical studies.

## Author contributions

ST and JF designed the work. ST wrote the first draft. Both authors contributed to the article and approved the submitted version.
