# Supplementary material for: Adverse effects of empathy and cognitive inflexibility on social trauma
Source: Front Psychol. 2023 May 26;14:1090297. doi: 10.3389/fpsyg.2023.1090297 (PMC10250643; doi:10.3389/fpsyg.2023.1090297)
Supplement: Supplementary file 1 [file Table_1.docx]

**Adverse Effects of Empathy and Cognitive Inflexibility on Social Trauma**

**Table S1. Glossary of key terms**

| Empathy | Ability to understand and share others’ feelings. |
| --- | --- |
| Affective empathy (AffEMP) | Ability to share the positive and negative feelings of another person. AffEMP can induce disproportionate affective intrusion of other people’s distress, which triggers traumatic experiences (Branson, 2019). |
| Cognitive empathy (CogEMP) | Ability to obtain accurate knowledge of the contents of another person’s mind (Zaki et al., 2009; Tei et al., 2020), which involves perspective-taking to infer others’ feelings and thinking. Altered CogEMP can augment cognitive bias and traumatic experiences (Mazza et al., 2015; Couette et al., 2020). |
| Self-other processing | Selective inhibition and activation of self and other representations to illuminate the experience of self and infer others’ experience. These processes involve the self-other distinction and overlap, which may prompt CogEMP and AffEMP to support complex adaptive (flexible) social behaviors. |
| Cognitive flexibility | A skill of self-control that enables adaptation to new, changing, or unplanned events. Flexibility helps shift avenues of thought or activity to perceive and process information in different ways. Shifts of this type also highlight alternative options or ideas to better grasp interpersonal situations (Uddin, 2021). Cognitive flexibility implies the readiness to selectively switch between mental processes and adjust to meet complicated or stressful circumstances without adherence to any particular perspective or belief (Fujino et al., 2017). Fundamentally, because cognitive flexibility facilitates adaptive responses to complex social situations, it may also prompt coping with traumatic experiences, related distress, and self-blame (Reich et al., 2021; Zimmer-Gembeck, 2021; Würtz, 2022; Wojcik et al., 2022). Cognitive flexibility may also alleviate trauma-related experiences (Neuner, 2022; Hamburger et al., 2021) by modulating the negative effects of empathy (i.e., empathic distress and empathic inaccuracy; Chikovani et al., 2015 Greenberg et al., 2018). |
| Social cognition | Ability to perceive, process, and comprehend social information. This frequently requires empathy and flexibility to prompt understanding and predict our own mental states or beliefs, and those of others, as well as being aware that they may be different. The impairment of social cognition may take part in the functional disability of individuals with PTSD (Couette et al., 2020). |

**References**

Branson, D.C. (2019). Vicarious Trauma, Themes in Research, and Terminology: A Review of Literature. *Traumatology* 25(1)**,** 2-10.

Chikovani, G., Babuadze, L., Iashvili, N., Gvalia, T., and Surguladze, S. (2015). Empathy costs: Negative emotional bias in high empathisers. *Psychiatry Res* 229(1-2)**,** 340-346. doi: 10.1016/j.psychres.2015.07.001.

Couette, M., Mouchabac, S., Bourla, A., Nuss, P., and Ferreri, F. (2020). Social cognition in post-traumatic stress disorder: A systematic review. *Br J Clin Psychol* 59(2)**,** 117-138. doi: 10.1111/bjc.12238.

Fujino, J., Tei, S., Jankowski, K. F., Kawada, R., Murai, T., & Takahashi, H. (2017). Role of spontaneous brain activity in explicit and implicit aspects of cognitive flexibility under socially conflicting situations: a resting-state fMRI study using fractional amplitude of low-frequency fluctuations. *Neuroscience*, *367*, 60-71.

Greenberg, D.M., Baron-Cohen, S., Rosenberg, N., Fonagy, P., and Rentfrow, P.J. (2018). Elevated empathy in adults following childhood trauma. *PLoS One* 13(10)**,** e0203886. doi: 10.1371/journal.pone.0203886.

Hamburger, A. (2021). The Complexity of Social Trauma Diagnosis and Intervention. In: Hamburger, A., Hancheva, C., Volkan, V.D. (eds) Social Trauma – An Interdisciplinary Textbook. Springer, Cham. https://doi.org/10.1007/978-3-030-47817-9_6

Mazza, M., Tempesta, D., Pino, M.C., Nigri, A., Catalucci, A., Guadagni, V., et al. (2015). Neural activity related to cognitive and emotional empathy in post-traumatic stress disorder. *Behav Brain Res* 282**,** 37-45. doi: 10.1016/j.bbr.2014.12.049.

Neuner, F. (2022). Physical and social trauma: towards an integrative transdiagnostic perspective on psychological trauma that involves threats to status and belonging. *Clinical psychology review*, 102219.

Reich, C. M., McKnight, K., Sacks, S. A., Farahid, N., Mulzon, T., Pegel, G., & Jamieson, J. (2023). Types of trauma-related blame following interpersonal trauma. Psychological Trauma: Theory, Research, Practice, and Policy, 15(2), 287–294. [https://doi.org/10.1037/tra0001139](https://psycnet.apa.org/doi/10.1037/tra0001139)

Tei, S., Kauppi, J.P., Jankowski, K.F., Fujino, J., Monti, R.P., Tohka, J., et al. (2020). Brain and behavioral alterations in subjects with social anxiety dominated by empathic embarrassment. *Proc Natl Acad Sci U S A* 117(8)**,** 4385-4391. doi: 10.1073/pnas.1918081117.

Uddin, L.Q. (2021). Brain Mechanisms Supporting Flexible Cognition and Behavior in Adolescents With Autism Spectrum Disorder. *Biol Psychiatry* 89(2)**,** 172-183. doi: 10.1016/j.biopsych.2020.05.010.

Würtz, F., Krans, J., Blackwell, S.E. (2022). Using Cognitive Bias Modification-Appraisal Training to Manipulate Appraisals about the Self and the World in Analog Trauma. *Cognitive Therapy and Research* 46**,** 232–246. doi: <https://doi.org/10.1007/s10608-021-10257-x>.

Wojcik, K.d. (2022). Maladaptive Schemas and Posttraumatic Stress Disorder Symptom Severity: Investigating the Mediating Role of Posttraumatic Negative Self-Appraisals among Patients in a Partial Hospitalization Program. *Journal of Aggression, Maltreatment & Trauma* 31(3)**,** 322-338. doi: 10.1080/10926771.2021.1994496.

Zaki, J., Weber, J., Bolger, N., and Ochsner, K. (2009). The neural bases of empathic accuracy. *Proc Natl Acad Sci U S A* 106(27)**,** 11382–11387. doi: 10.1073/pnas.0902666106 0902666106 [pii].

Zimmer-Gembeck, M.J. (2021). Coping flexibility: Variability, fit and associations with efficacy, emotion regulation, decentering and responses to stress. *Stress Health* 37(5)**,** 848-861. doi: 10.1002/smi.3043.
